# Immunomodulation with IL-7 and IL-15 in HIV-1 infection

**DOI:** 10.1016/j.jve.2023.100347

**Published:** 2023-09-05

**Authors:** Jesper D. Gunst, Nilu Goonetilleke, Thomas A. Rasmussen, Ole S. Søgaard

**Affiliations:** a Department of Clinical Medicine, Aarhus University, Aarhus, Denmark; b Department of Infectious Diseases, Aarhus University Hospital, Aarhus, Denmark; c Department of Microbiology & Immunology, University of North Carolina, Chapel Hill, North Carolina, USA; d Department of Medicine, University of North Carolina, Chapel Hill, North Carolina, USA

## Abstract

Immunomodulating agents are substances that modify the host immune responses in diseases such as infections, autoimmune conditions and cancers. Immunomodulators can be divided into two main groups: 1) immunostimulators that activate the immune system such as cytokines, toll-like receptor agonists and immune checkpoint blockers; and 2) immunosuppressors that dampen an overactive immune system such as corticosteroids and cytokine-blocking antibodies. In this review, we have focussed on the two primarily T and natural killer (NK) cell homeostatic cytokines: interleukin-7 (IL-7) and -15 (IL-15). These cytokines are immunostimulators which act on immune cells independently of the presence or absence of antigen. *In vivo* studies have shown that IL-7 administration enhances proliferation of circulating T cells whereas IL-15 agonists enhance the proliferation and function of NK and CD8^+^ T cells. Both IL-7 and IL-15 therapies have been tested as single interventions in HIV-1 cure-related clinical trials. In this review, we explore whether IL-7 and IL-15 could be part of the therapeutic approaches towards HIV-1 remission.

## 1 Immunomodulation in the presence or absence of antigen

HIV-1 disease is a chronic condition treatable with antiretroviral therapy (ART). Due to the persistence of a stable latent virus reservoir that is unaffected by ART, lifelong treatment is needed for people with HIV-1.^1^To achieve HIV-1 remission, defined as sustained ART-free virological control, this latent HIV-1 reservoir needs to be eradicated, reduced, silenced and/or contained by potent HIV-1-specific immune responses. In this review, we will focus on two immunostimulatory homeostatic cytokines, IL-7 and IL-15, which both act on immune cells independently of the presence or absence of antigen. Other reviews focus on interventions that rely on the presence of HIV antigen like broadly neutralizing anti-HIV-1 antibodies (bNAbs) and bispecific molecules.^2^^,^^3^

In HIV-1 disease, immunomodulatory interventions are usually administered during suppressive ART, where the majority of HIV-1 proviruses remains in a latent state and HIV-1 antigen burden is low. However, recent studies have begun to explore immunomodulatory interventions given during the viremic state^4^ or prior to and at the start of an ART interruption (ATI) period.^5^ It is important to acknowledge that immunomodulators are likely to have differential pharmacodynamic properties depending on the degree of antigen exposure and/or responsiveness of the responder cells, making the interventional setting a key determinant of the immunomodulatory impact on the desired outcome.^6^, ^7^, ^8^

## 2 Interleukin-7 and -15

Both IL-7 and IL-15 are cytokines produced by a variety of cells, and they are crucial for homeostasis, activation and function of natural killer (NK) and T cells, as well as maintenance of memory T cell responses.^9^, ^10^, ^11^ IL-7 and IL-15 are members of the four α-helix bundle family of cytokines, which also includes another four interleukins: IL-2, IL-4, IL-9 and IL-21. These cytokines share the common cytokine receptor γ-chain (γ_c_ also known as CD132), while only IL-2 and IL-15 share the other part of the heterodimeric receptor, the IL-2/IL15Rβ-chain also known as CD122. IL-15 first binds to the membrane-bound IL-15Rα that is highly expressed by monocytes and dendritic cells and is then *trans*-presented in a cell-cell contact-dependent manner to responder cells expressing the IL-2/IL15Rβ-γ_c_ heterodimer. IL-15 signalling can also occur by *cis*-presentation or through soluble IL-15Rα.^10^^,^^11^ IL-7 binds and signals via IL-7Rα (also known as CD127) as the last part of the heterodimeric receptor, and exists in membrane-bound and soluble forms. The IL-7Rα is mainly expressed on the least differentiated T cell subsets, while the IL-2/IL15Rβ is primarily expressed on the more differentiated ones.^10^^,^^12^^,^^13^ Both IL-7 and IL-15 signal downstream through Janus kinases (JAK)/signal transducer and activator of transcription proteins (STAT) pathways.^9^^,^^11^

Modifications have improved the pharmacokinetic profile of recombinant human IL-7 and IL-15, especially cytokine half-life.^11^^,^^14^^,^^15^ A long-acting IL-7 was made by fusing recombinant human IL-7 with a hybrid immunoglobulins D/G4 Fc (rhIL-7-hyFc).^16^ Two key modifications (heterodimerization and superagonist) of IL-15 have increased binding ability to IL-2/IL15Rβ-γ_c_ and thus immunostimulatory activity 1) A recombinant, heterodimeric form of IL-15 bound to IL-15Rα (het-IL-15)^17^; and 2) A superagonist mutein containing amino acid substitutions, including IL-15N72D,^18^ and a superagonist fusion where IL-15N72D is bound to a sushi domain of IL-15Rα fused with immunoglobulin G1 Fc, also known as N-803 (previously ALT-803).^19^

Under normal homeostatic conditions without lymphopenia, plasma levels of IL-7 and IL-15 are tightly regulated, being either undetectable or very low by standard assays.^10^^,^^20^ In studies of people with HIV-1, plasma levels of IL-7 and IL-15 correlated negatively with CD4^+^ T cell counts and positively with plasma HIV-1 RNA levels.^13^^,^^20^, ^21^, ^22^, ^23^ In turn, elevated plasma IL-7 and IL-15 levels are associated with downregulation of their cellular receptors.^7^^,^^12^ The effects of homeostatic cytokines on CD4^+^ T cell proliferation in HIV-1 disease depends on the setting: 1) During initial untreated disease; the levels of homeostatic cytokines are high reflecting the ‘lack’ of consumption due to high turnover of CD4^+^ T cells, followed by 2) A progressively decrease of the levels of homeostatic cytokines and increase of CD4^+^ T cells counts after ART initiation,^13^^,^^20^, ^21^, ^22^, ^23^ but the responding receptor expression on T cells is not fully restored within 2 years.^12^^,^^24^ In an earlier study using single structured ATI as a way to augment autologous HIV-1-immunity, individuals with delayed/absent viral rebound during ATI had significantly increased levels of plasma IL-15 prior to and during ATI compared to individuals with rapid viral rebound.^25^ In another study, the expression of IL-17 mRNA in and IL-15Rα on monocytes was higher among long-term non-progressors than progressors.^7^ In cancer therapy, repeated doses of IL-7 administration was shown to increase numbers of circulating T cells in a dose-dependent manner,^26^ while the different IL-15 therapies increased proliferation and function of circulating NK and CD8^+^ T cells.^9^ Collectively, these findings suggest that there is a therapeutic rationale for investigating treatment with IL-7 and IL-15 in HIV-1.^27^^,^^28^

## 3 *In vitro* and *ex vivo* effects of IL-7 or IL-15

The cell types and tissues assayed as well as the results from *in vitro* and *ex vivo* experiments of IL-7 or IL-15 are summarized in Table 1. We focus on the immune-stimulating effect of IL-7 or IL-15 administration on virus-specific CD8^+^ T cells as well as NK cells, and briefly describe their latency reversing potential.

**Table 1:** Summary of the *in vitro* and *ex vivo* effects of IL-7 or IL-15 therapies.

	Year	Reference	Set-up	Cells		Analyses		Results
Interleukin 7	1993	Moran PA. et al. ^47^	*ex vivo*	CD8^−^ PBMCs		Cell proliferation assay		Enhanced proliferation
						p24 ELISA in supernatant		Induced viral replication
	1994	Carini C. et al. ^30^	*ex vivo*	PBMCs and CD8^+^ T cells		Cell proliferation assay		Enhanced proliferation
				PBMCs		Killing of target cell		Enhanced killing
				CD8^+^ T cells				
	1996	Smithgall MD. et al. ^45^	*ex vivo*	CD8^−^ PBMCs		Cell proliferation assay		Enhanced proliferation
						p24 ELISA in supernatant		Induced viral replication
						Tat expression in cells		Induced viral transcription
				CD8^+^ PBMCs		p24 ELISA in supernatant		No viral replication
	1999	Chêne L. et al. ^49^	*in vitro*	CD4^+^ T cells		p24 ELISA in supernatant		Induced viral replication
	1999	Unutmaz D. et al. ^51^	*in vitro*	Resting (CD69-HLA-DR-) CD3^+^ T cells		IL-7 pretreated HIV exposure		Infection
				Naive (CD45RA + CD45RO-) CD3^+^ T cells				
				Resting (CD69-HLA-DR-) memory (CD45RO+) CD4^+^ T cells		CCR5 expression		Induced CCR5 expression
	2002	Ducrey-Rundquist O. et al. ^103^	*in vitro*	CD4^+^ T cells		IL-7 pretreated HIV exposure		Infection (memory CD45RA-CD45RO + more susceptible than naive CD45RA + CD45RO-)
	2002	Scripture-Adams DD. et al. ^48^	*ex vivo* of infected humanized mice	PBMCs		p24 ELISA in supernatant		Induced viral replication
				CD4^+^ T cells				
						Expression of virus-encoded reporter		Induced transcription
			*ex vivo*	Memory (CD45RO-) T cells		T cell activation	CD25	Enhanced activation
				Effector (CD45RA-) T cells			CD69	No change
				Resting (HLA-DR-CD25^−^CD69) memory (CD45RO-) T cells		Phenotypic distribution		Induced CD45RA expression; distribution towards the memory compartment
				Resting (HLA-DR-CD25^−^CD69) effector (CD45RA-) T cells				
	2002	Steffens CM. et al. ^104^	*in vitro*	Naive (CD45RA + CD45RO-) CD3^+^ T cells		Cell proliferation assay		Enhanced proliferation
						Intracellular Ki67 staining		Enhanced proliferation (CD8^+^ T cells > CD4^+^ T cells)
						HIV exposure followed by IL-7		No infection
						IL-7 pretreated HIV exposure		Infection
						CCR5 expression		No induced CCR5 expression
	2005	Wang F-X. et al. ^46^	*in vitro*	1G5 cell line		LTR transactivation assay		Modest effect on inducing transcription
			*ex vivo*	CD8^−^ PBMCs		p24 ELISA in supernatant		Induced viral replication (10/18)
				Resting (CD25-HLA-DR-) CD4^+^ T cells				Induced viral replication (5/11)
						T cell activation	CD25	Enhanced activation
							HLA-DR	No change
							CD69	No change
						Intracellular Ki67 staining		Enhanced proliferation
	2007	Vassena L. et al. ^55^	*ex vivo*	CD4^+^ T cells		p24 ELISA in supernatant		No viral replication
				PBMCs	CD4^+^ and CD8^+^ T cells	Intracellular annexin V and caspase 3 staining		Induced antiapoptotic effect
						Intracellular Ki67 staining		Enhanced proliferation
	2009	Chomont N. et al. ^52^	*ex vivo*	CD4^+^ T cells		Phenotypic distribution		Minimal effect; distribution towards the memory compartment
						Intracellular Ki67 staining		Enhanced proliferation
	2011	Bosque A. et al. ^54^	*in vitro*	Naive (CCR7+CD45RA+) differentiated to CM (CCR7+CD27^+^) CD4^+^ T cells		Phenotypic distribution of TCM		No change
						Intracellular Ki67 and p24 staining		Enhanced proliferation, but no viral transcription
	2013	Vandergeeten C. et al. ^53^	*ex vivo*	CD4^+^ T cells		Ultrasensitive RT-PCR in supernatant		Minimal viral replication
						Intracellular Ki67 staining		Enhanced proliferation
						T cell activation	HLA-DR	Enhanced activation
						Ultrasensitive RT-PCR in supernatant		Induced viral replication
				Resting (HLA-DR-CD69^−^CD25^−^) memory (CD45RA-) CD4^+^ T cells				Minimal viral replication
	2016	Coiras M. et al. ^50^	*in vitro*	CD4^+^ T cells		Viral reverse transcripts		Induced viral transcription
						Proviral integration		Induced the level of integrated DNA
	2016	Jones RB. et al. ^37^	*in vitro*	CD4^+^ T cells		p24 ELISA in supernatant		Induced viral replication
						CD8^+^ T-cell clone: Recognition assay		Facilitated recognition of latently infected cells
	2016	Younes SA. et al. ^29^	*ex vivo*	CD8^+^ T cells		Intracellular Ki67 staining		Enhanced proliferation
						Intracellular granzyme-B staining		Enhanced cytotoxicity
Interleukin 15	1995	Seder RA. et al. ^111^	*ex vivo*	PBMCs		Cell proliferation assay		Enhanced proliferation
					CD4^+^ T cells	IFNγ ELISA in supernatant		Minor effect on cytotoxicity
	1996	Kanai T. et al. ^33^	*ex vivo* of infected NHPs	PBMCs		Cell proliferation assay		Enhanced proliferation
					CD8^+^ T cells	Killing of target B-LCL cell line		Enhanced killing
			*ex vivo*	PBMCs				
						Cell proliferation assay		Enhanced proliferation
	1997	Chehimi J. et al. ^38^	*ex vivo*	PBMCs		Cell proliferation assay		Enhanced proliferation
						Cell apoptosis assay		Inhibited apoptosis
						IL-15 pretreated HIV exposure		Infection depending on strain
						p24 ELISA in supernatant in anti-CD3-stimulated		Minimal viral replication
						NK cell-mediated killing of target K562 cell line		Enhanced killing
				NK cells		mRNA expression of granzyme B and perforin		Enhanced cytotoxicity
			*in vitro*	U1 and ACH2 cell lines		RT activity in supernatant		No viral replication
	1997	Loubeau M. et al. ^39^	*ex vivo*	PBMCs		Killing of target K562 cell line		Enhanced killing
						ADCC assay using CEM-NK-resistant cells		Enhanced ADCC
	1999	Naora H. et al. ^112^	*ex vivo*	Memory (CD45RO+) T cells		T cell activation	CD69	Enhanced activation
				Effector (CD45RO-) T cells		Intracellular 7-AAD staining		Inhibited apoptosis
	2003	Mueller YM. et al. ^31^	*ex vivo*	CD4^+^ T cell subsets (CD45RA ± CD62L±)		Anti-CD3 T cell activation	CD69	Enhanced activation
				CD8^+^ T cell subsets (CD45RA ± CD62L±)				
				PBMCs		IFNγ ELISA in supernatant		No change
						Anti-CD3-induced IFNγ ELISA in supernatant		Enhanced cytotoxicity
				Effector memory (CD45^−^CD62L-) CD8^+^ T cells		Intracellular annexin V staining		Inhibited apoptosis
	2003	Mueller YM. et al. ^34^	*ex vivo*	Effector memory (CD45^−^CD62L-) HIV-1-specific CD8^+^ T cells		Intracellular annexin V staining		Inhibited apoptosis
				HIV-1-specific CD8^+^ T cells				
						Anti-CD3-induced T cell activation	CD69	Enhanced activation
						T cell activation		
						MFI of IFNγ intracellular staining		Enhanced cytotoxicity
						Frequency of IFNγ-producing cells		No change
						Killing of target C1R-A2 cells		Enhanced killing
	2016	Jones RB. et al. ^37^	*in vitro*	CD4^+^ T cells		p24 ELISA in supernatant		Induced viral replication
						CD8^+^ T-cell clone: Recognition assay		Facilitated recognition of latently infected cells
	2016	Younes SA. et al. ^29^	*ex vivo*	CD8^+^ T cells		Intracellular Ki67 staining		Enhanced proliferation
						Intracellular granzyme-B staining		Enhanced cytotoxicity
	2018	Garrido C. et al. ^40^	*ex vivo*	NK cells		Killing of target cells		Enhanced killing
						Surface CD107a expression		Enhanced degranulation
						Intracellular IFNγ		Enhanced cytotoxicity
						ADCC assay using CEM-NK-resistant cells		Enhanced ADCC
						Latency clearance assay		Removed number of p24 HIV + wells
	2019	Fisher L. et al. ^41^	*ex vivo*	NK cells		ADCC assay using CEM-NK-resistant cells		Enhanced ADCC
						Phenotypic distribution		CD56_bright_ NK cells increased
						NK cell activation	HLA-DR	Enhanced activation
							CD69	
						Intracellular perforin and granzyme B staining		Enhanced cytotoxicity
	2022	Covino DA. et al. ^42^	*ex vivo*	NK cells		Killing of target K562 cell line		Enhanced killing
						NK-mediated killing of activated CD4^+^ T cells		
			*in vitro*	Latently infected CD4^+^ T cells		p24 ELISA in supernatant		Induced viral replication
ALT-803	2014	Jones RB. et al. ^35^	*ex vivo*	CD8^+^ T cells		IFNγ ELISPOT assay		Enhanced cytotoxicity
	2015	Seay K. et al. ^36^	*ex vivo*	PBMCs		Intracellular perforin and granzyme B staining		Enhanced cytotoxicity
						Surface CD107a expression		Enhanced degranulation
						Killing of target K562 cell line		Enhanced killing
						Killing of activated ACH2 cells		
	2016	Jones RB. et al. ^37^	*in vitro*	CD4^+^ T cells		p24 ELISA in supernatant		Induced viral replication
						CD8^+^ T-cell clone: Recognition assay		Facilitated recognition of latently infected cells
			*ex vivo*			Viral RNA in supernatant		Induced viral replication
						T cell activation	CD69	Enhanced activation
						Cell proliferation assay		No change
						Total HIV DNA/10^6^ CD4^+^ T cells		No change
						CD8^+^ T-cell clone: Recognition assay		Enhanced killing

Studies are listed chronologically. Two studies are not mentioned in the text, but included in this table [111–112]. Abbreviations: ADCC; antibody-dependent cellular cytotoxicity, B-LCL; lymphoblastoid B-cell line, CCR5; C–C chemokine receptor type 5, ELISA; enzyme-linked immunosorbent assay, ELISPOT; enzyme-linked absorbent spot,IFN; inteferon, IL; interleukin, LTR; long terminal repeat, NK; natural killer, PBMCs; peripheral blood mononuclear cells, PCR; polymerase chain reaction, RT; reverse transcription, 7-AAD; 7-Aminoactinomycin D.

*Immune-modulatory effects*: IL-7 stimulation of peripheral blood mononuclear cells (PBMCs) *ex vivo* enhanced virus-specific CD8^+^ T cell cytotoxicity^29^ and function^30^ (against influenza and cytomegalovirus). IL-15 stimulation *ex vivo* of isolated CD8^+^ T cells enhanced activation, but this was only observed in the most differentiated subsets.^31^ During untreated HIV-1 infection, activated CD8^+^ T cells are more susceptible to apoptosis^32^ *ex vivo*, which can be inhibited by IL-15.^31^ IL-15 stimulation of PBMCs also increased numbers,^33^ activation status^34^ and functional capacity^33^ of HIV-1-specific CD8^+^ T cells, whilst their susceptibility to apoptosis again was reduced.^34^ IL-15 stimulation of pre-activated PBMCs enhanced the frequency of HIV-1-specific T cells^31^ and non-specific IFNγ secretion in the supernatant^34^ several fold compared to IL-15 stimulation without pre-activation. *Ex vivo* stimulation with the IL-15 superagonist N-803 also enhanced HIV-1-specific T-cell cytotoxicity^35^^,^^36^ and function.^36^^,^^37^ IL-15 stimulation enhanced cytotoxic and functional capacities of NK cells^38^, ^39^, ^40^, ^41^, ^42^ and antibody-dependent cellular cytotoxicity.^39^, ^40^, ^41^ After IL-15 stimulation, NK cells had higher expression of the activation receptor NKG2D and cytotoxicity receptor NKp30^39^^,^^40^^,^^42^ (also seen after *in vivo* N-803 administration^43^), while expression of the cytotoxicity receptor NKp46 was decreased.^40^^,^^42^ IL-15 stimulation and NKG2D engagement were needed for vaccine-primed- K cells to control HIV-1 in autologous CD4^+^ T cells.^44^

*Latency reversing potential:* IL-7 stimulation of different cell types have been shown to reverse latency,^37^^,^^45^, ^46^, ^47^, ^48^, ^49^, ^50^, ^51^ but studies later have shown that latently infected CD4^+^ T cells were maintained, in part, through IL-7-induced homeostatic proliferation,^50^^,^^52^^,^^53^ even without reversing latency.^53^, ^54^, ^55^ Additionally, IL-7 stimulation of T cells resulted in a shift of subset distribution towards the memory compartment,^48^^,^^52^ which supports the critical role of IL-7 for generation of long-lived memory T cells.^56^ IL-15 may also work as latency-reversing agent.^37^^,^^38^^,^^42^ More importantly, IL-7- and IL-15-primed latently infected cells to increased CD8^+^ T cell recognition and killing, which is an important finding since latently infected cells after stimulation with other latency-reversing agents seems to be resistant to killing.^37^^,^^40^^,^^57^^,^^58^ IL-15 in combination with other latency-reversing agents have been shown to abrogate each other's latency -reversing effects, although the combination of IL-15 and a protein kinase C agonist potentiated viral (re)activation and showed sustain NK cell function.^42^

In summary, *ex vivo* studies indicate that IL-7 stimulation may maintain or even expand the size of the HIV-1 reservoir due to homeostatic proliferation of infected CD4^+^ T cells. In contrast to IL-7, IL-15 stimulation appears to enhance cellular responses against HIV-1 *in vitro* and *ex vivo* without expanding the size of the HIV-1 reservoir.

## 4 Effects of IL-7 and IL-15 in HIV-1 animal models

Of note, in murine^59^, ^60^, ^61^, ^62^ and HNP models,^63^, ^64^, ^65^ the use of IL-7 or IL-15 as vaccine adjuvants enhanced the immune responses to HIV/SIV/SHIV vaccines, but this vaccine adjuvant effect could not be repeated in humans.^66^ IL-15-adjuvanted SIV vaccination prior to SIV challenge preserved the CD4^+^ T cell numbers in the tissue to a greater extent than vaccination without IL-15 as adjuvant.^67^ Further, some non-human primates (NHPs) primed with both TLR agonists and IL-15 prior to SIV vaccination were protected again SIV infection.^68^

*Immune-modulatory effects:* IL-7 administration among simian immunodeficiency virus (SIV)-infected NHPs enhanced proliferation^69^, ^70^, ^71^, ^72^ and activation^69^^,^^71^ of CD4^+^ and CD8^+^ T cells leading to a transient increase in numbers^69^, ^70^, ^71^, ^72^ (Table 2). The increase in absolute T cell numbers occurred in both the naïve and central memory subsets, which also mainly express IL-7Rα.^69^, ^70^, ^71^, ^72^, ^73^ The expression of IL-7Rα on these cells was transiently downregulated following IL-7 administration.^70^^,^^71^ The proliferative effects were more sustained with repeated than single IL-7 administration.^73^ IL-7 stimulation also enhanced HIV-1-specific CD8^+^ T cell cytotoxicity,^74^ and lymphadenopathy occurred following subcutaneous (SC) IL-7 injection due to cell migration.^69^^,^^70^

**Table 2:** Summary from animal studies using IL-7 or IL-15 therapies.

	Year	Reference	Animals (n)/isolate/dosing		Cells (in blood unless stated)	Analyses		Results
Interleukin 7	2003	Fry TJ. et al. ^70^	ART-treated rhesus macaques (n = 8)		CD4^+^ and CD8^+^ T cells	Intracellular Ki67 staining		Enhanced proliferation
						T cell activation	CD69, HLA-DR or CD25	No activation
			SIVmac251			Absolute cell counts		Increased cell numbers
					Naive (CD45RA + CD27^+^) CD4^+^ and CD8^+^ T cells			
			SC rhIL-7 daily at 100 μg/kg for 9 days		Lymph node	Size		Lymphadenopathy
					Plasma (during suppression)	Viral load		No changes
	2003	Nugeyre M-T. et al. ^69^	ART naïve rhesus macaques (n = 2)		CD4^+^ and CD8^+^ T cells	Intracellular Ki67 staining		Enhanced proliferation
						T cell activation	HLA-DR	Enhanced activation
			SIVmac251			Absolute cell counts		Increased cell numbers
					Naive (CD45RA + CD62L+) CD4^+^ and CD8^+^ T cells			
			SC rhIL-7 twice daily at 40 μg/kg for 21 days		Lymph node	Size		Lymphadenopathy
						Viral load	In situ hybridization	No changes
					Plasma (during viremia)		Real time RT-qPCR	
	2006	Beq S. et al. ^71^	ART-treated rhesus macaques (n = 4)		CD4^+^ and CD8^+^ T cells	Intracellular Ki67 staining		Enhanced proliferation
						T cell activation	HLA-DR or CD25	Enhanced activation
			SIVmac251			Absolute cell counts		Increased cell numbers
					Naive (CD45RA^bright^CD62L+) CD4^+^ and CD8^+^ T cells			
			SC rsIL-7 every 2 days at 100 μg/kg for 26 days		Memory (CD45RA^−^/_low_CD62L±) CD4^+^ and CD8^+^ T cells			
					Plasma (during suppression)	Viral load		No changes
	2010	Leone A. et al. ^73^	ART-treated rhesus macaques (n = 5)		CD4^+^ and CD8^+^ T cells	Intracellular Ki67 staining (naive and CM) Absolute cell counts (all)		Enhanced proliferation Increased cell numbers
			SIVmac239		Naive (CD28^int^CD95^low^CCR7^int^CCR5-) CD4^+^ and CD8^+^ T cells			
			SC rsIL-7 at 30 mg/kg in three dosing regimens: one, two, or three weekly; up to 9 doses		CM (CD95^hi^CD28^hi^CCR7+CCR5-) CD4^+^ and CD8^+^ T cells			
					EM (CD95hiCD28-CCR7-CCR5dim+) CD4^+^ T cells			
					TrM (CD28^hi^CCR5+ and/or CCR7-) CD4^+^ T cells			
	2017	Steele AK. et al. ^72^	ART-treated rhesus macaques (n = 5)		CD4^+^ and CD8^+^ T cells	Intracellular Ki67 staining Absolute cell counts		Enhanced proliferation Increased cell numbers
			SIVmac239		Naive (CD28^int^CD95^low^CCR7^int^CCR5-) CD4^+^ and CD8^+^ T cells			
			Three cycles of SC rsIL-7 at 30 mg/kg weekly for 3 weeks followed by 2 weeks between each cycle		CM (CD95^hi^CD28^hi^CCR7+CCR5-) CD4^+^ T cells			
					EM (CD95hiCD28-CCR7-CCR5dim+) CD8^+^ T cells			
					TrM (CD28^hi^CCR5+ and/or CCR7-) CD8^+^ T cells			
	2007	Hryniewicz A. et al. ^65^	ART suppressed rhesus macaques (SIVmac251)		Plasma (during ATI)	Viral load	RT-PCR	Reduced viral set-point (mock + IL-7, SIV vaccine ± IL-7 or ± IL-15)
					CD4^+^ T cells	Absolute cell counts		No further increase by SIV vaccine, IL-7 or IL-15
			Mock or SIV vaccine (n = 3 + 8) x 3 +		Viral reservoir	IUPM in PBMCs		Increased in size following IL-15 as opposed to IL-7 or no cytokine
			SC IL7 at 100 μg/kg (n = 2 + 5) every third day; total 8 doses (x 2 along first 2 immunizations)		Naive (CD28 + CD95^−^) CD4^+^ and CD8^+^ T cells	Intracellular Ki67 staining		Proliferation following SIV vaccine + IL-7
Interleukin 15					EM (CD95 + CD95^+^) CD4^+^ and CD8^+^ T cells			Proliferation following SIV vaccine + IL-15
			or		SIV-specific CD8^+^ T cells	Frequency	Tetramer staining	No effect of adding IL-7 or IL-15
			SC rsIL-15 at 10 μg/kg (n = 2 + 5) twice weekly: total 6 doses (x 3)			Flowcytometry; IFN-γ and TNF-α		
						Flowcytometry; frequency of CD107a (degranulation)		Increased after addition of IL-15
	2005	Mueller YM. et al. ^75^	ART naïve rhesus macaques (n = 3 + 3)		CD8^+^ T cells	Intracellular Ki67 staining		Enhanced proliferation
					CD4^+^ T cells	Absolute cell counts		No changes
			SIVmac251		NK and CD8^+^ T cells			Increased cell numbers (high dose)
					EM (CD45RA ± CD62L-) CD8 + T cells			
			SC rsIL-15 at 10 (low) or 100 (high) μg/kg twice weekly for 4 weeks		SIV gag-specific CD8^+^ T cells	ELISpot assay; IFN-γ-secretion		No changes
					Plasma (during viremia)	Viral load	RT-PCR	No changes
	2008	Mueller YM. et al. ^76^	ART naïve rhesus macaques (n = 6)		Plasma (during viremia)	Viral load	RT-PCR	Increased viral set-point
					SIV gag-specific CD8^+^ T cells	Absolute cell counts		Increased during intervention, but decreased afterwards
			SIVmac251		CD8^+^ T cells	T cell activation	HLA-DR	Decreased activation
					CM (CD45RA-CD62L+) CD4^+^ T cells	Frequency		Increased long-term memory compartment
					EM (CD45RA-CD62L-) CD4^+^ T cells			Decreased short-term memory compartment
			SC rsIL-15 at 100 μg/kg twice weekly for 4 weeks		SIV gag-specific CD8^+^ T cells	Flowcytometry; IFN-γ and TNF-α		No changes
					NK cells	Killing of target K562 cell line		No changes
	2011	Lugli E. et al. ^80^	ART naïve rhesus macaques		Plasma (during suppression)	Viral load	RT-PCR	Delayed time to suppression compared to ART alone (IL-15 ± ART)
			SIVmac251		CD8^+^ T cells	T cell activation	HLA-DR	Faster decreased of activation (ART + IL-15)
			ART initiation + IL-15 (n = 6), ART (n = 6) or IL-15 alone (n = 4)		CD4^+^ and CD8^+^ T cells	Absolute cell counts		Accelerated T cell recovery after IL-15 + ART
			rsIL-15 at 80 μg/kg twice weekly for 6 weeks		Plasma (during ATI)	Viral load	RT-PCR	No delayed time to viral rebound
	2018	Watson DC. et al. ^81^	ART naïve rhesus macaques (n = 15)		CM (CD28highCD95+) + EM (CD28^−^CD95^+^) CD4^+^ T cells	Intracellular Ki67 staining Frequency		Enhanced proliferation Increased compartment
			SHIV (clade B or C)		EM (CD28^−^CD95^+^) CD8^+^ T cells (blood + lymph node)			
			SC human (11) and macaque (4) hetIL-15 at step-dosing (2–64 μg/kg) over 2 weeks; total 6 doses		NK (CD3^−^CD16^+^) cells			
					GzmB + CD8^+^ T cells (lymph node)	Absolute cell counts		Increased numbers in the follicles
					Plasma and lymph node (viral burden)	Viral load	qPCR, RT-PCR	Decreased viral burden
	2021	McCann CD. et al. ^77^	A HIV participant-derived xenograft (PDX) mouse model		Plasma (during ATI)	Viral load	RT-PCR	Transient reductions (IL-15-engineered CD8^+^ T cells)
								Increased viral set-point (IL-15 superagonist)
			ART naïve	HIV_JR-CSF_	CD4^+^ T cells	T cell activation	CD25	Increased activation (IL-15 superagonist)
			IL-15-engineered CD8^+^ T cells (2 doses: n = 6; 1 dose n = 7) or CD8^+^ T cells + SC IL-15 superagonist (n = 6) at 0.2 mg/kg three weekly doses at day of infection				CD69	
					CD4^+^ T cells	Absolute cell counts		Reduced CD4^+^ T cell depletion
					CD8^+^ T cells			Expansion of CD8^+^ T cells
ALT-803	2018	Ellis-Connell AL. et al. ^85^	ART naïve rhesus macaques (n = 4)		Plasma (during viremia)	Viral load	RT-PCR	Decreased viral burden
			SIVmac239		CD4^+^, CD8^+^ T and NK cells	Absolute cell counts		Increased cell numbers
			SC ALT-803 at 100 μg/kg weekly; total 4 doses		EM (CD28^−^CD95^+^) CD8 + T cells			Increased cell numbers
			(n = 3) received a second and third cycle after 2 and 35 week break from first cycle		Plasma (during viremia)	Viral load	RT-PCR	Decreased viral burden (during first and third cycle)
					CD8^+^ T and NK cells	Absolute cell counts		Increased cell numbers (memory subset)
					SIV-specific CD8^+^ T cells	Flowcytometry; IFN-γ, TNF-α and CD107a		No consistent increased in cytotoxicity
					CD8^+^ T cells	T cell exhaustion	PD-1 and CD39	Increased numbers of EM cells
	2018	Webb GM. et al. ^84^	ART naïve rhesus macaques (n = 4)		CD4^+^ and CD8^+^ T cells	Absolute cell counts		Increased cell numbers (memory subset)
			SIVmac251			Intracellular Ki67 staining		Enhanced proliferation (memory subset)
					CD16^+^ NK cells			Enhanced proliferation
			IV ALT-803 at 6 μg/kg at day 0, 7, and 14 followed by 100 μg/kg at day 49		SIV-specific CD8^+^ T cells			
						Frequency/absolute count	Tetramer stain	Increased frequent and count
					Plasma (during viremia)	Viral load		No changes
			ART naïve rhesus macaques (n = 5)		CD8^+^ T cells (lymph node)	Frequency		Increased in frequency
			SIVmac251		SIV-specific CD8^+^ T cells (lymph node)		Tetramer stain	
			IV ALT-803 at 100 μg/kg single dose		Viral reservoir (lymph node)	Cell-associated SIV DNA in CD4^+^ T cells		Decreased number of SIV-producing cells
	2020	McBrien JB. et al. ^82^	ART suppressed rhesus macaques (n = 7)		CD8^+^ T cells (blood and lymph node)	Frequency and absolute cell count		Increased in frequency and cell numbers
			SIVmac239		CD4^+^, CD8^+^ T and NK cells	Intracellular Ki67 staining		Enhanced proliferation
			ART initiated 8 weeks post-infection and continued for 12 months prior to SC 100 ALT-803 at 100 μg/kg once weekly; total 4 doses		CD4^+^ T cells	CCR5 expression	Flowcytometry	Induced CCR5 expression
					Plasma (during suppression)	Viral load	RT-PCR	No changes
					Plasma (during ATI)			No delayed time to viral rebound
	2020	Webb GM. et al. ^82^	ART naïve rhesus macaques (n = 6)		CD8^+^ T and NK cells	Absolute cell counts		Increased cell numbers (memory subset)
			SHIV_SF162P3_			Intracellular Ki67 staining		Enhanced proliferation
			ART initiation (2 weeks post-infection) followed (20 weeks) by SC ALT-803 at 100 μg/kg biweekly total 4 doses		Plasma (during suppression)	Viral load	RT-PCR	No changes
					CD8^+^ T and NK cells (lymph node)	Frequency		Increased in frequency
					SHIV-specific CD8^+^ T cells (lymph node)			
					CD3^−^CD159a NK cells (lymph node)			
					Viral reservoir (lymph nodes, spleen, colon)	Viral load	SHIV-DNA/CD4+ T cells	Decreased viral burden (only in spleen)
					Plasma (during ATI)	Viral load	RT-PCR	No delayed time to viral rebound

Studies are listed chronologically. Abbreviations: ATI; analytical treatment interruption, CCR5; C–C chemokine receptor type 5, ELISPOT; enzyme-linked absorbent spot, IFN; inteferon, IL; interleukin, IUMP, infectious units per million cells; NK; natural killer, PBMCs; peripheral blood mononuclear cells, PCR; polymerase chain reaction, rs; recombinant simian, RT; reverse transcription, SC; subcutaneous; SHIV; Simian-Human Immunodeficiency Virus, SIV; Simian Immunodeficiency Virus.

IL-15 administration among viremic NHPs only led to modest increases in CD8^+^ T cell and NK numbers^6^^,^^75^^,^^76^ as opposed to what was observed in SIV-uninfected NHPs (reviewed elsewhere^27^) as well as *ex vivo* IL-15 stimulation of pre-activated PBMCs as described above .^31^^,^^34^ In one of the studies, IL-15 administration led to increased viral set-point (as seen in a HIV mice model^77^) and accelerated disease progression.^76^ So the modest effect seen on CD8^+^ T and NK cells of additional exogen IL-15 administration during untreated infection could be due to already high levels of endogen IL-15^78^ as well as short half-life of responder cells.^27^ Whether or not IL-15 stimulation during the viremic phase of HIV-1 infection might provide superior effects on T cell function remains to be determined; however, one concern would be that of excessive CD8^+^ T cell activation, which is known to be associated with worse outcomes in people with HIV-1.^79^

IL-15 administration at ART initiation among SIV-infected NHPs increased the number of CD8^+^ T cells.^80^ More importantly, following IL-15 administration, the virus-specific CD8^+^ T cell proliferated,^80^ but no effect was observed on IFNγ cytotoxicity^75^^,^^80^ or control during ATI.^80^ In non-progressors and ART-suppressed SIV-infected NHPs, IL-15 increased the proliferative activity of T cells^6^^,^^81^ and enhanced frequency of T effector memory cells expressing granzyme B.^81^ N-803 administration was also tested among ART-treated virally-suppressed NHPs, which generally confirmed the findings described above .^82^^,^^83^

Studies have also tested whether N-803 could benefit virus-specific immunity among more favourable clinical phenotypes (SIV controllers) in the absence of ART.^84^^,^^85^ N-803 administration enhanced number of NK and CD8^+^ T cells,^83^, ^84^, ^85^ which correlated with the observed decreases in plasma viral loads.^84^^,^^85^ After N-803 and het-IL-15 administration, there was increased proliferation of NK and CD8^+^ T cells.^81^^,^^83^, ^84^, ^85^ There are conflicting findings on whether the cytotoxicity of virus-specific CD8^+^ T cells were increased^81^ or not^85^ following N-803 administration. The majority of the NK and CD8^+^ T cells that increased in numbers following N-803 expressed CD16^+^ and had effector memory phenotype, respectively.^81^^,^^83^, ^84^, ^85^ Virological control during ATI was not observed among SIV/SHIV-infected NHPs after N-803 administration.^82^^,^^83^ Of note, prior to N-803 administration, SIV-specific CD8^+^ T cells primarily localized in the extrafollicular space of lymph nodes, but after N-803 administration the numbers of SIV-specific CD8^+^ T cells significantly increased within B-cell follicles.^84^ Migration of CD8^+^ T cells to the lymph nodes occurred via upregulation of CXC chemokine receptor 5 (CXCR5).^84^ The expression of IL-2/IL15Rβ-y_c_ on central memory CD8^+^ T cells transiently declined during weekly N-803 administration, but was somewhat restored by deferring administration,^85^ thus spacing administrations seems optimal due to refractoriness.^6^^,^^75^^,^^85^, ^86^, ^87^, ^88^ Multiple administrations of N-803 led to increased PD-1 expression on CD8^+^ T cells, which might be due to activation rather than exhaustion.^85^

In two HIV mice models IL-15-primed CD8^+^ T cells showed enhanced *in vivo* activity on initial viremia when given at the day of infection, but administration of an IL-15 superagonist led to increased plasma viral set-point.^77^ In the other model, N-803 administration 3 days after infection induced NK cell-mediated inhibition of acute infection.^36^ Thus, interventional approaches within the first days of infection in these models does not mirror what is feasible in people with HIV-1.

*Latency reversing potential:* No latency reversal effect of IL-7 on plasma viremia has been observed.^69^, ^70^, ^71^ Following N-803 administration, plasma viral loads decreased indicating that the *in vivo* latency reversal effect of N-803 might be masked due to antiviral effector functions,^37^^,^^82^^,^^84^^,^^85^ which also explains the diverse findings from the other studies using IL-15 therapies.^6^^,^^75^^,^^80^^,^^81^^,^^83^

Several studies have assessed the effect of IL-15 therapies on the size of the viral reservoir with mixed results (Table 2), as it was found to either decrease,^81^^,^^83^ remain unchanged^82^^,^^84^ or increase.^65^

In summary, in multiple studies across several animal models, IL-7 administration increased absolute T cell numbers, and N-803 administration increased the total numbers of NK and CD8^+^ T cells, with spaced administration of more than one week being most optimal. Despite these proliferative effects of IL-15, more information is needed on the HIV-1-specific effects as well as antiviral functions of the effector cells following the interventions as there was no apparent impact of IL-15 on virological control among animals that had interrupted ART.

## 5 Effects of IL-7 and IL-15 therapy in people with HIV-1

*Immune-modulatory effects:* Four clinical trials have tested IL-7 among people with HIV-1 on suppressive ART.^89^, ^90^, ^91^, ^92^ The maximum tolerated dose (MTD) was 30 μg/kg with single subcutaneous (SC) injections^89^ and 20 μg/kg MTD of repeated injections.^91^^,^^92^ The administration of IL-7 was overall well tolerated with the most reported adverse events being injection-site reactions and transient elevations of liver function enzymes.^89^, ^90^, ^91^, ^92^, ^93^ The results were overall similar to the findings from NHPs: IL-7 administration enhanced proliferation as measured by the expression of Ki67 on CD4^+^ and CD8^+^ T cells leading to increased cell number^89^, ^90^, ^91^; mainly in the central memory (CD45RA-CCR7+^90^^,^^91^^,^^93^ or CD45RA-CD62L-^89^), but also the naïve (CD45RA + CCR7+)^90^^,^^91^^,^^93^ subsets, confirming prevalent IL-7Rα expression on these subsets. Frequencies of HIV-1-specific CD8^+^ T cells were unchanged after IL-7 administration, but one study has observed a trend towards enhanced proliferation as measured by Ki67 expression.^89^ IL-7 administration did not affect T cell subset distribution, PD-1 frequencies or IL-7Rα expression.^89^

Several clinical cancer trials have tested the safety and effect of IL-15 therapies. Pharmacokinetics was dependent on the exact IL-15 compound that was tested as well as the route of administration. IL-15 administration SC had a longer half-life due to a slower release,^94^ but intravenous (IV) administration resulted in higher plasma concentrations, which explain the difference in SC versus IV toxicity. Of note, a greater mean fold increase in circulating numbers of NK and CD8^+^ T cells were observed after SC compared to IV administration of N-803,^43^^,^^94^ and doses up to 20 μg/kg were tested IV without significant toxicities. In 2022, a phase 1 dose-escalation study of N-803 was conducted among 16 people with HIV-1 on suppressive ART.^95^ Five different dosing schemes were planned with three individuals per scheme receiving three doses once weekly. Two individuals received the lowest dose of 0.3 μg/kg IV, but due to data from cancer trials showing increased risk of a cytokine release syndrome with IV administration, the following doses were given as SC administration. The MTD was found to be 6.0 μg/kg, and of note no anti-N-803 antibodies have been observed in 6.0 μg/kg cohorts.^94^^,^^96^^,^^97^ Every individual experienced Grade 3 injection site erythema and 22 (65%) of the 34 injections were associated with adenopathy, but none of them were Grade 1. There were differences in pharmacodynamics at 6.0 μg/kg between cancer and HIV trials (Table 3), which have been ascribed to target-mediated drug disposition.^15^ In the HIV trial, three N-803 administrations increased the number of circulating NK over CD8^+^ T cells with enhanced proliferation as measured by Ki67 expression on both cell types. N-803 also increased expression of the activation markers, CD69 and HLA-DR on both NK and CD8^+^ T cells but the study could not demonstrate increased HIV-1-specific T cell responses.

**Table 3:** Pharmacodynamics of ALT-803 after 6.0 μg/kg subcutaneous administration.

	Cancer trial^43^		HIV-1 trial^95^	
Number of participants	8		3	
T_max_ in hours, median (range)	35	(0.5–56)	8	(8-24)
C_max_ in pg/mL	389	317	54	(29–165)
	Mean	±sd	Median	(range)
Half-life in hours	22		36	(8-24)
	Mean^a^		Median	(range)

Abbreviations: C_max_; maximum serum concentration, T_max_; time of C_max_, sd; standard deviation.

a Only analyzed in one patient.

*Latency reversing potential:* Mixed results were seen on whether IL-7 could work as a latency-reversing agent, but if plasma viremia increased this was ascribed to activation of already transcriptional active cells rather than reactivation of *de novo* viral transcription^53^^,^^98^ as observed with other latency-reversing agents.^99^ IL-7-induced expansion of the HIV-1 reservoir was confirmed.^50^^,^^52^^,^^54^^,^^90^, ^91^, ^92^, ^93^ In the phase 1 clinical trial using N-803, there was evidence of a latency-reversal effect, in that 91% and 100% of the individuals had detectable plasma viral loads and increased HIV-1 mRNA transcription in memory CD4^+^ T cells during the interventional period, respectively.^95^

The impact of N-803 on the latent HIV-1 reservoir was investigated using two assays. The frequency of memory CD4^+^ T cells that could be activated to viral transcription was significantly reduced over the 6-months course of the trial (*P* =<0.001). By contrast, the level of intact proviral HIV-1 DNA per million CD4^+^ T cells measured by the intact proviral DNA assay (IPDA) actually increased over the interventional period (*P* = 0.098). The levels of the defective proviral HIV-1 DNA per million CD4^+^ T cells did not change over the interventional period, so the authors have speculated that the intact reservoir increased due to the expansion rather than the infection of new cells, but only integration-site analyses can ultimate clarify this point.

In summary, IL-7 and N-803 have overall shown to be safe and well-tolerated among people with HIV-1 on suppressive ART. Whereas the overall number of T cells increased with IL-7 therapy, including those that were latently infected, N-803 primarily led to increases in CD8^+^ T and NK cells. Additional trials are needed to address whether more potent cellular responses against HIV-1 are developed following the interventions and their effect on ART-free virological control during an ATI (NCT04808908, NCT04505501).

## 6 Future directions for IL-7 or IL-15 therapy in HIV-1

**Fig. 1:**
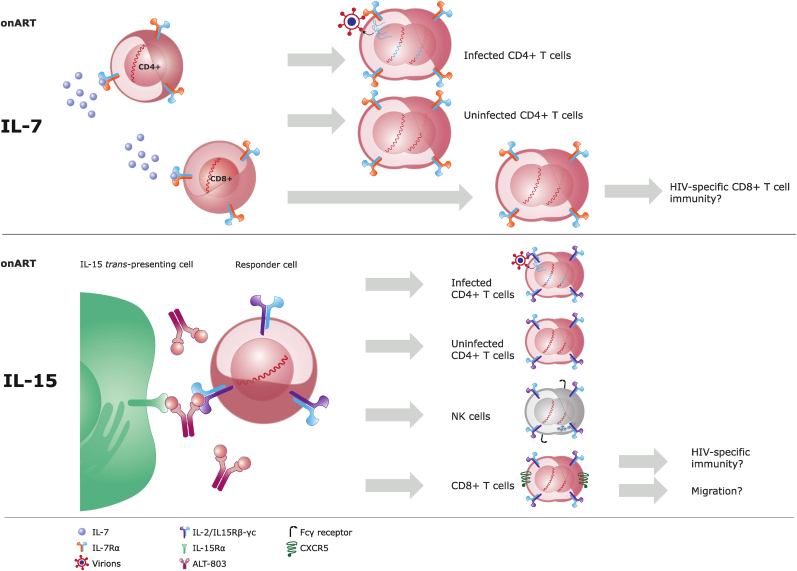
Effects of IL-7 and IL-15 therapies *in vivo* during suppressive antiretroviral threapy (ART). (a) Illustration of the effects of IL-7 administration during suppressive ART (on ART). IL-7 binds to the IL-7Rα receptor, here on the CD4^+^ and CD8^+^ T cells (mainly naïve and central memory subsets). Cells undergo homeostatic proliferation with expansion of T cell counts. In the CD4^+^ T cell compartment, both infected and uninfected cells proliferate leading to the expansion of the HIV-1 reservoir. During the proliferation of the infected CD4^+^ T cells, some degree of latency reversal occurs and a transient increase in plasma HIV-1 RNA levels can be observed. The CD8^+^ T cell compartment also proliferates, which might also expand the HIV-1-specific CD8^+^ T cells. The expression of IL-7Rα is downregulated following IL-7 stimulation of T cells.^22^^,^^37^^,^^42^ (b) Illustration of the effects of IL-15 superagonist N-803 administration during suppressive ART (on ART). N-803 binds to membrane IL-15Rα on a *trans*-presenting cell and in a cell-cell contact-dependent manner to responder cells expressing IL-2/IL15Rβ-γ_c_. Upon stimulation with N-803 the responder cells undergo homeostatic proliferation. As seen with administration of IL-7, during proliferation of the infected CD4^+^ T cells some degree of latency reversal occurs and a transient increase in plasma HIV-1 RNA levels can be observed. In the CD4^+^ T compartment, both infected and uninfected cells expand. The NK cells and CD8^+^ effector memory (EM) T cells also expand. Expansion of the CD8^+^ EM T cells might also lead to the expansion of the HIV-1-specific CD8^+^ T cells as well as upregulation of the expression of CXCR5 leading to tissue migration.

Modern ART regimens are highly effective at suppressing plasma viremia, achieving undetectable levels within months of treatment. However, restoration of CD4^+^ T cell counts takes considerably longer, in some cases years. Central to this immune reconstitution is endogenous IL-7 and IL-15 production which regulates CD4^+^ T cell homeostasis (Fig. 1), and thus, could interventions with IL-7 or IL-15 help restoration of CD4^+^ T cells? There are two issues with the homeostatic cytokines that should be considered prior to any intervention. 1) The cytokine-induced CD4^+^ T cell expansion of the HIV-1 reservoir,^50^^,^^100^ which might be overcome by making the interventional homeostatic cytokines CD8-targeted, but then the restoration of the CD4^+^ T cell compartment would be missed, or by simultaneously inhibiting cell-intrinsic anti-apoptosis pathways to diminish infected CD4^+^ T cells^101^^,^^102^; and 2) The increased susceptibility to infection^103^^,^^104^ by enhanced CCR5 expression on CD4^+^ T cells.^51^^,^^82^^,^^100^^,^^105^ Importantly, a recent study found that IL-15 stimulation *in vitro* and in humanized mice promoted proliferation and survival of the HIV-1 target cell (CCR5 expressing CD4^+^ T cells), and furthermore the life span of infected CCR5-expressing CD4^+^ T cells was prolonged and their virus production was increased.^100^

IL-15 agonists could have a role in HIV-1 cure-related strategies. N-803 induced proliferation of NK and CD8^+^ T cells among ART-suppressed people with HIV-1,^95^ and enhanced migration of these cells to the tissues *in vivo*.^6^^,^^84^ The impact of N-803 on T cell migration and homing is important given that homeostatic proliferation of infected cells also occurs in tissues.^106^ Expression of IL-15Rα on responder cells are somewhat restored by ART, but inclusion of a TLR agonist could further induce IL-15Rα expression^67^ and thus enhance the effect of N-803. As stated in the introduction, the timing of immunotherapies relative to viremia versus viral suppression is currently being explored, but interventions with homeostatic cytokines in this setting seems unfit since levels are already elevated.

**Fig. 2:**
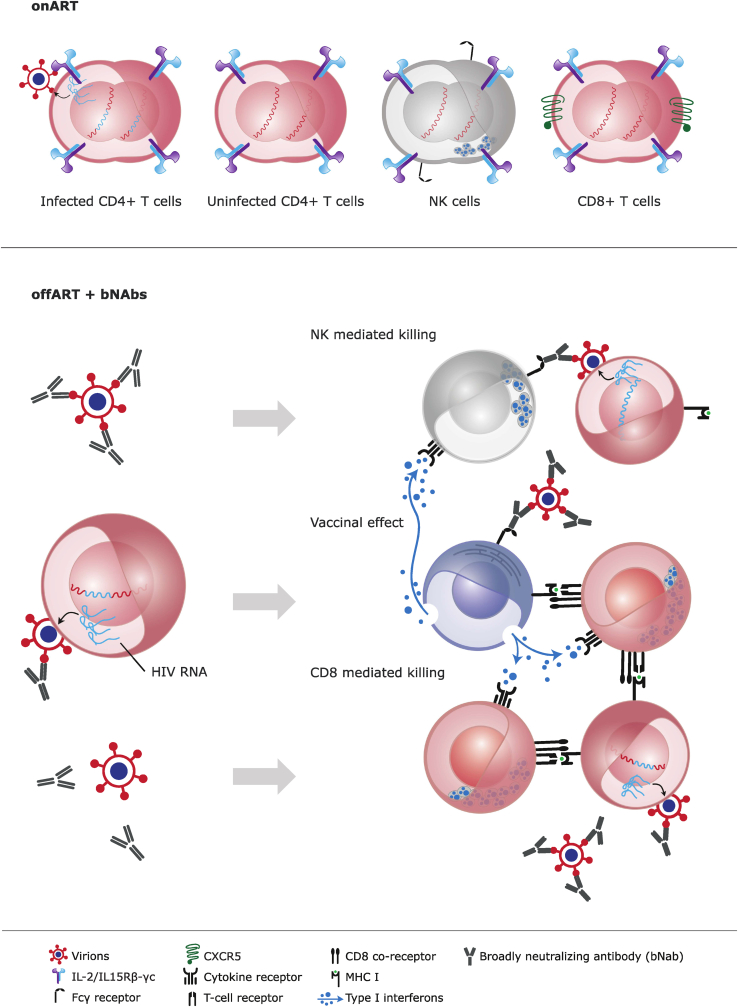
Effects of IL-15 stimulation on antiretroviral therapy (ART) and in a combinatorial approach with broadly neutralizing anti-HIV-1 antibodies (bNAbs) off ART. (a) Illustration of the effects of IL-15 superagonist N-803 administration during suppressive ART (on ART) as shown in Fig. 1. (b) Illustration of the effects of N-803 in combination with bNAbs prior to (NCT04340596) and into (NCT05245292) an analytical treatment interruption (ATI; off-ART). During ATI, infected CD4^+^ T cells with inducible HIV-1 reservoir might be (re)activated to initiated transcription/translation due to immune activation, which can lead to antibody-dependent cellular cytotoxicity by the NK and CD8^+^ T cells. Viral particles produced by the (re)activated infected CD4^+^ T cells can form bNAbs-antigen complexes that bind to plasmacytoid dendritic cells (pDCs). This cross presents viral antigens leading to the development of HIV-1-specific CD8^+^ T cell and enhanced killing of infected cells – a vaccinal effect.^5^

Despite some latency reversal effect of plasma viremia by N-803, available data indicates that more effective latency reversal is needed to decrease the HIV-1 reservoir.^107^ One interventional strategy to overcome reversal of latency is to pause ART during an ATI to let the virus rebound: Two clinical trials are testing this strategy with administration of N-803 in combination with two broadly neutralising antibodies (bNAbs) during suppressive ART and then conducting an ATI at a later time point (NCT04340596) or N-803 in combination with two bNAbs during suppressive ART, but administered 2 days prior to an ATI (NCT05245292) (Fig. 2). The *ex vivo* findings of enhanced antibody-dependent cellular cytotoxicity^39^, ^40^, ^41^ and enhanced expression of CD16 on NK cells in animal studies^81^^,^^83^, ^84^, ^85^ have not been confirmed among people with HIV-1, but in theory the combinatorial approach with bNAbs will result in killing of infected cells by different Fc-mediated mechanisms (Fig. 2). The presence of bNAbs at therapeutic levels will also limit the infection of new cells by direct neutralization of cell-free virions. Another interventional combination with N-803 could include an immune checkpoint inhibitor. A mathematical model based upon one of the NHP studies^85^ has shown that co-administration of an immune checkpoint inhibitor may improve N-803 efficacy,^108^ which has been confirmed in a phase 1b cancer trial.^97^ Other combinatorial approaches with N-803 being tested in cancer research are with antibodies,^109^ cell^110^ or gene therapy (NCT05618925, NCT04847466). Notably, as for other interventions tested in HIV-1 research,^99^ N-803 has also induced person-specific changes (NK cell functionality) in cancer trials moving curative interventions towards a personalized medicine approach.^43^

In conclusion, IL-7 has shown to be safe and well-tolerated among people with HIV-1 leading to increased absolute T cell numbers, including latently infected cells. Prior to future trials using IL-7, this expansion of the HIV-1 reservoir needs to be hindered. IL-15 therapies have also shown to be safe and well-tolerated among people with HIV-1, leading to increased total numbers of primarily CD8^+^ T and NK cells. Thus, IL-15 administration could be part of therapeutic approaches towards HIV-1 remission, but future trials should have broadened the immunological assessments on both the HIV-1 reservoir as well as antiviral responses: HIV-1-specific immunity and the effect on virological control during ATI. Since HIV-1 infection causes widespread dysregulation of the host immune system that is slow to recover, even after successful ART, the interventional timing is another important aspect to consider.

## Declaration of competing interest

The authors declare that they have no known competing financial interests or personal relationships that could have appeared to influence the work reported in this paper.

## Data Availability

No data was used for the research described in the article.
